# Supplementary figures and images for: Functional and structural deficiencies of Gemin5 variants associated with neurological disorders
Source: Life Sci Alliance. 2022 Apr 7;5(7):e202201403. doi: 10.26508/lsa.202201403 (PMC8989681; doi:10.26508/lsa.202201403)

Figure 2A

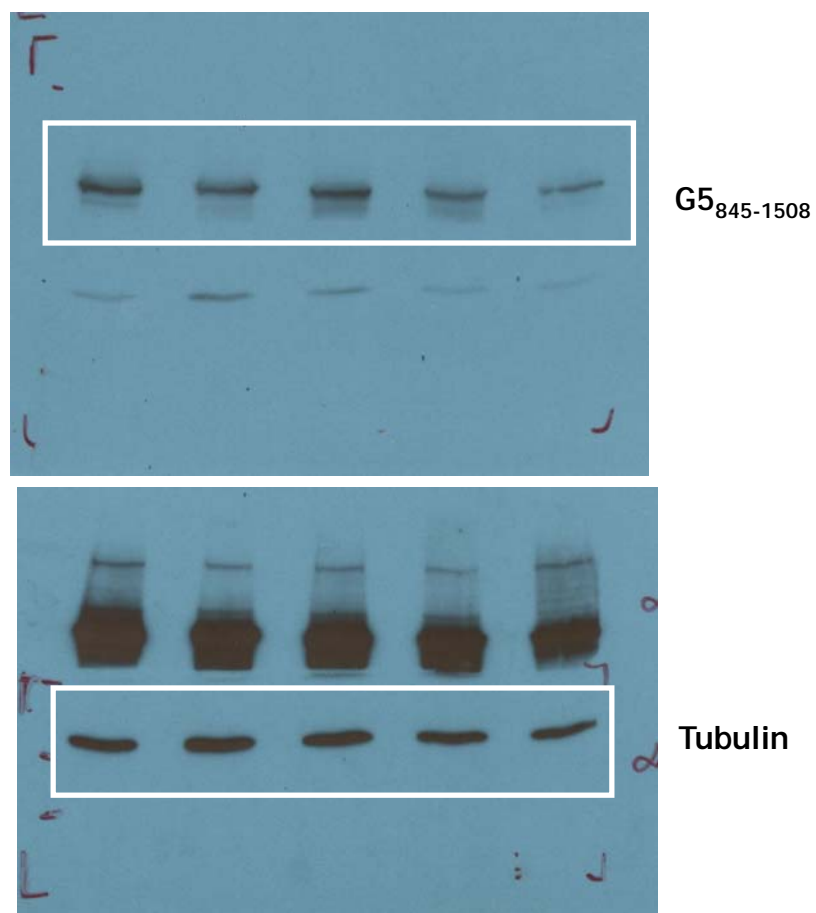

Figure 2B

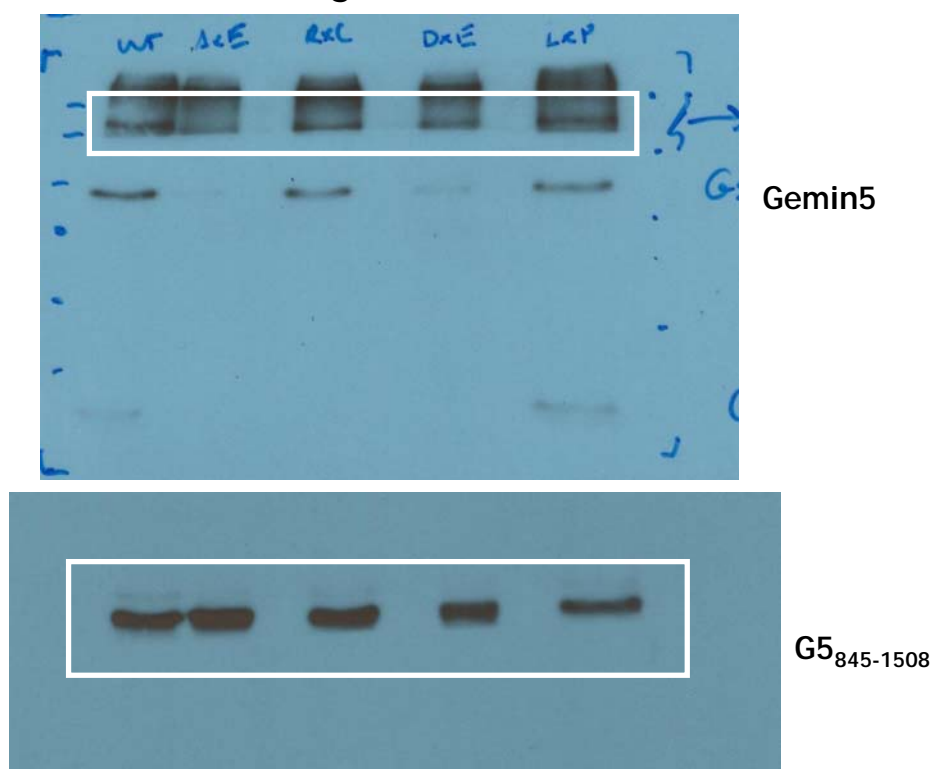

Supplement: Supplementary file 1 [file LSA-2022-01403_SdataF2.pdf]

Figure S3 A

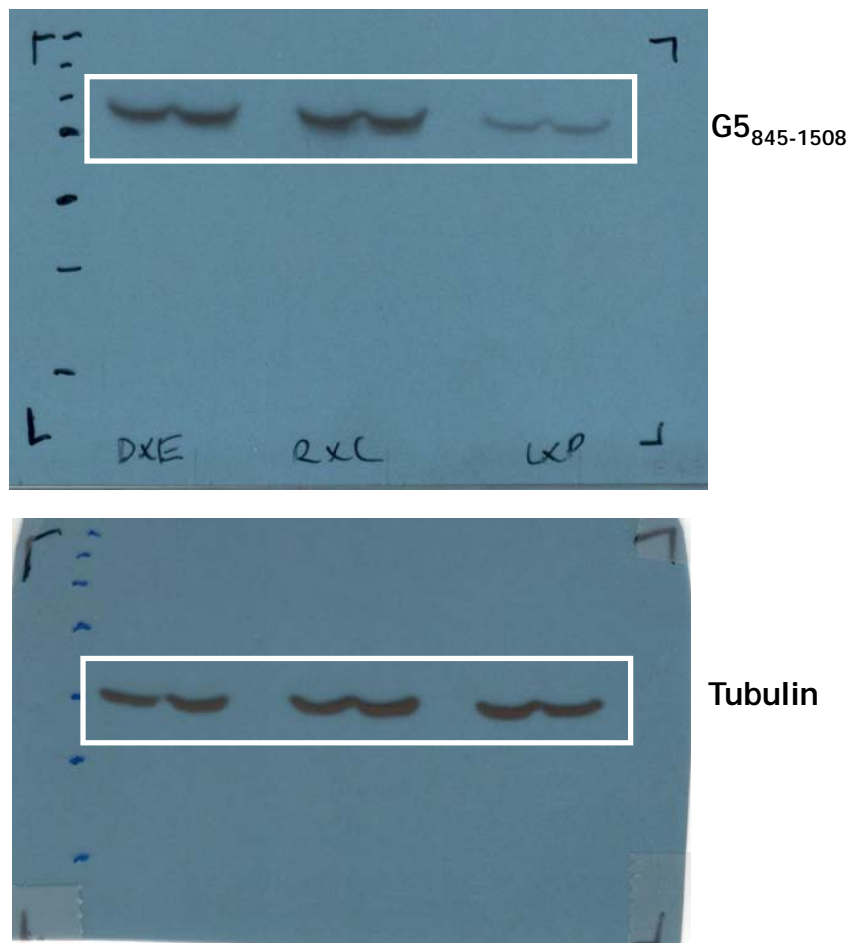

Figure S3 B

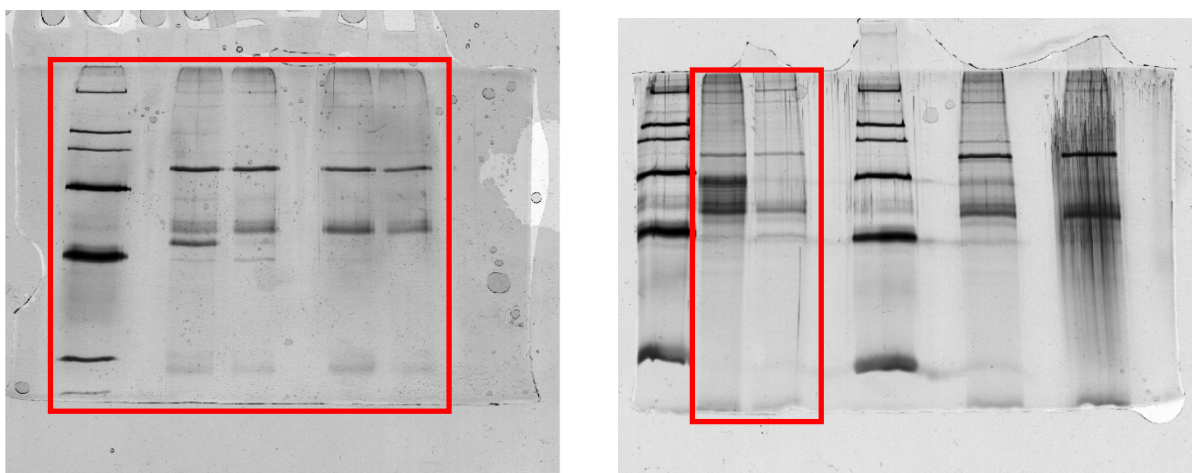

Supplement: Supplementary file 3 [file LSA-2022-01403_SdataFS3.pdf]

Figure 4A

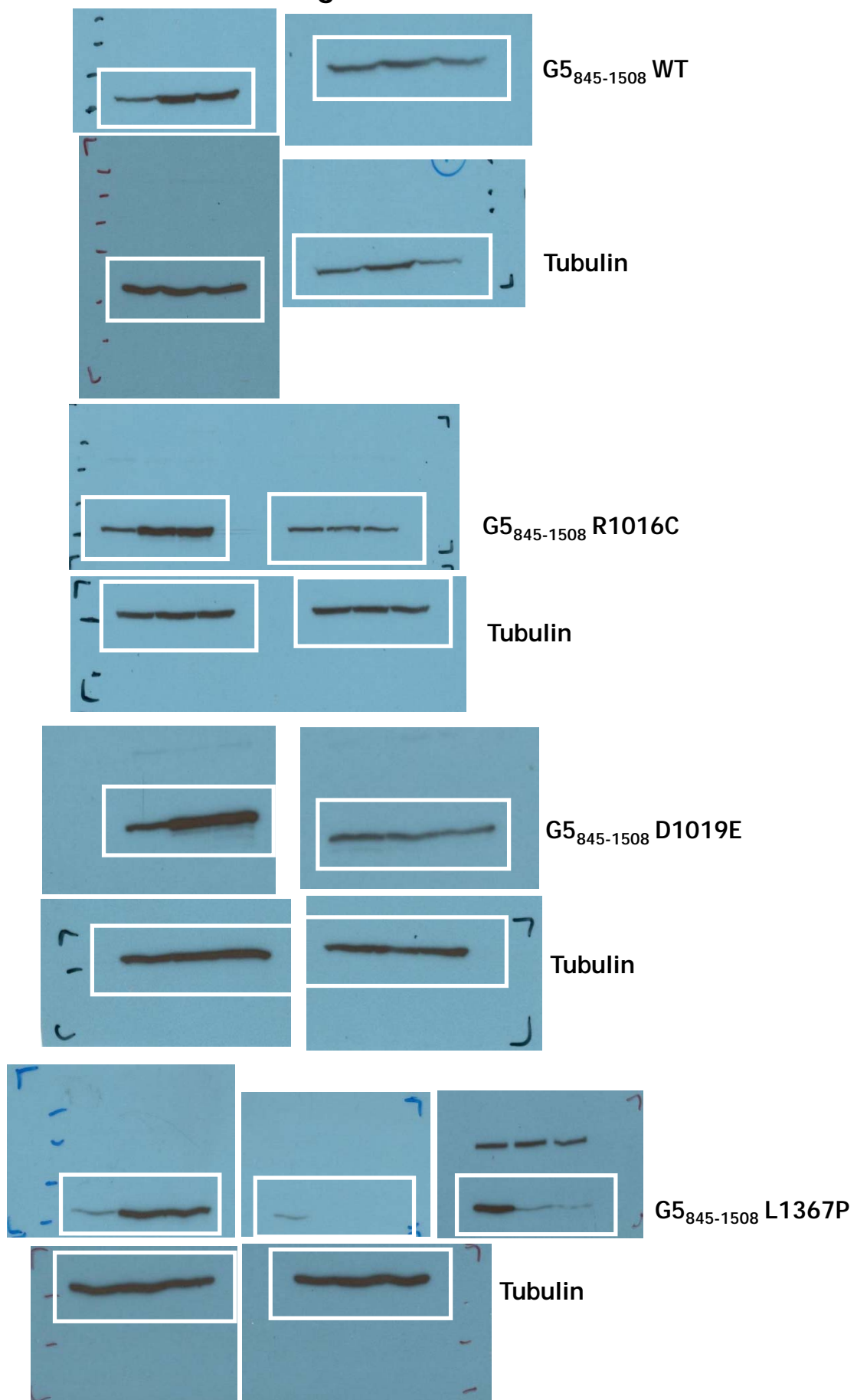

Supplement: Supplementary file 4 [file LSA-2022-01403_SdataF4.pdf]

Figure S4 A

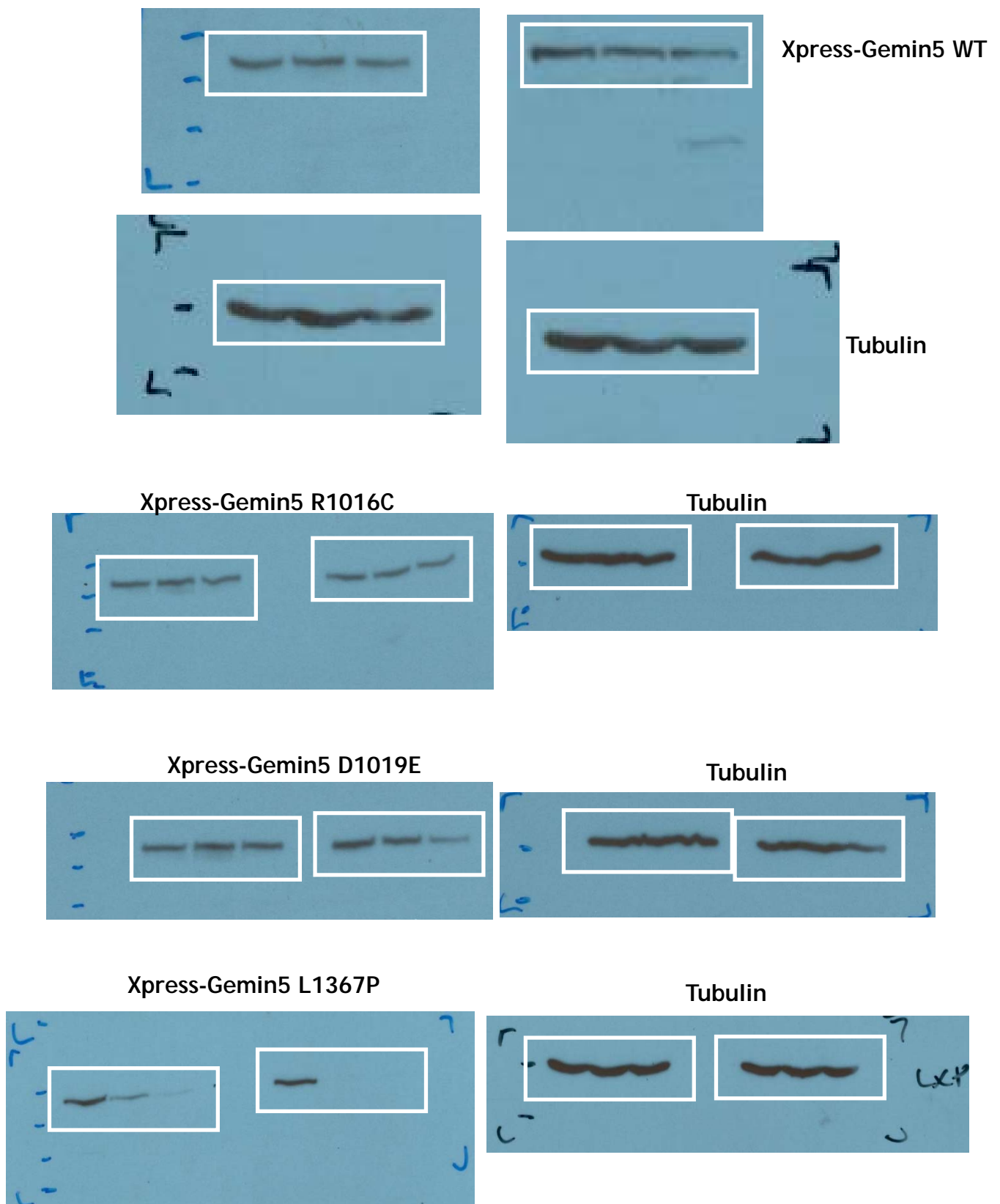

Supplement: Supplementary file 5 [file LSA-2022-01403_SdataFS4.pdf]

Figure 5A

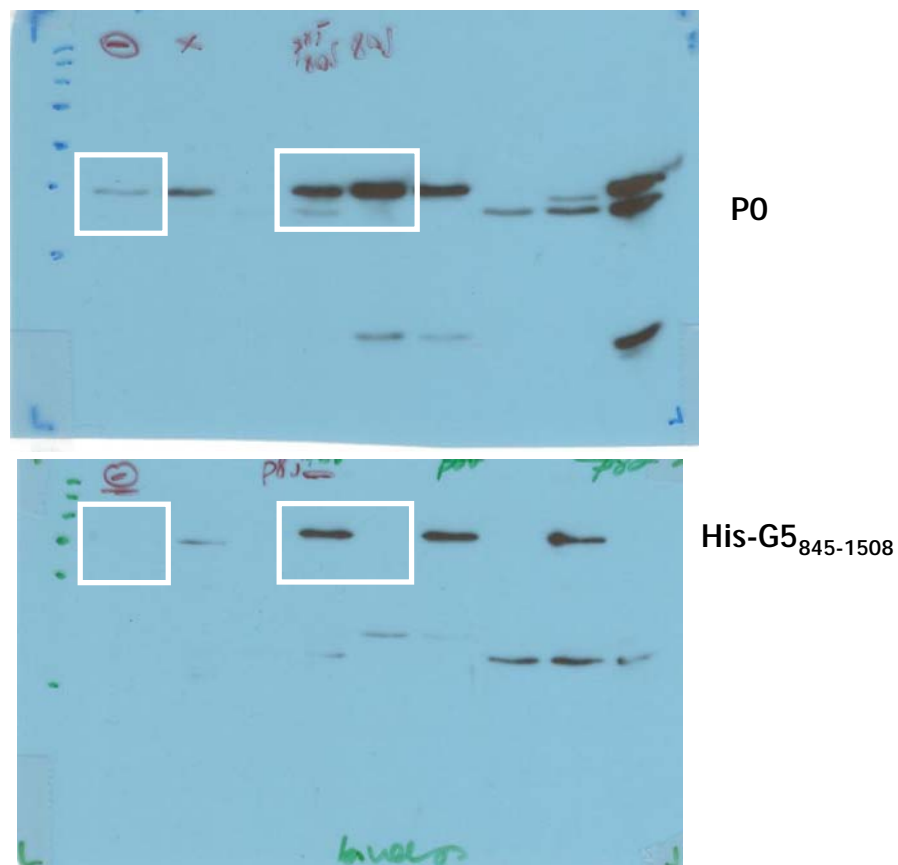

Figure 5B

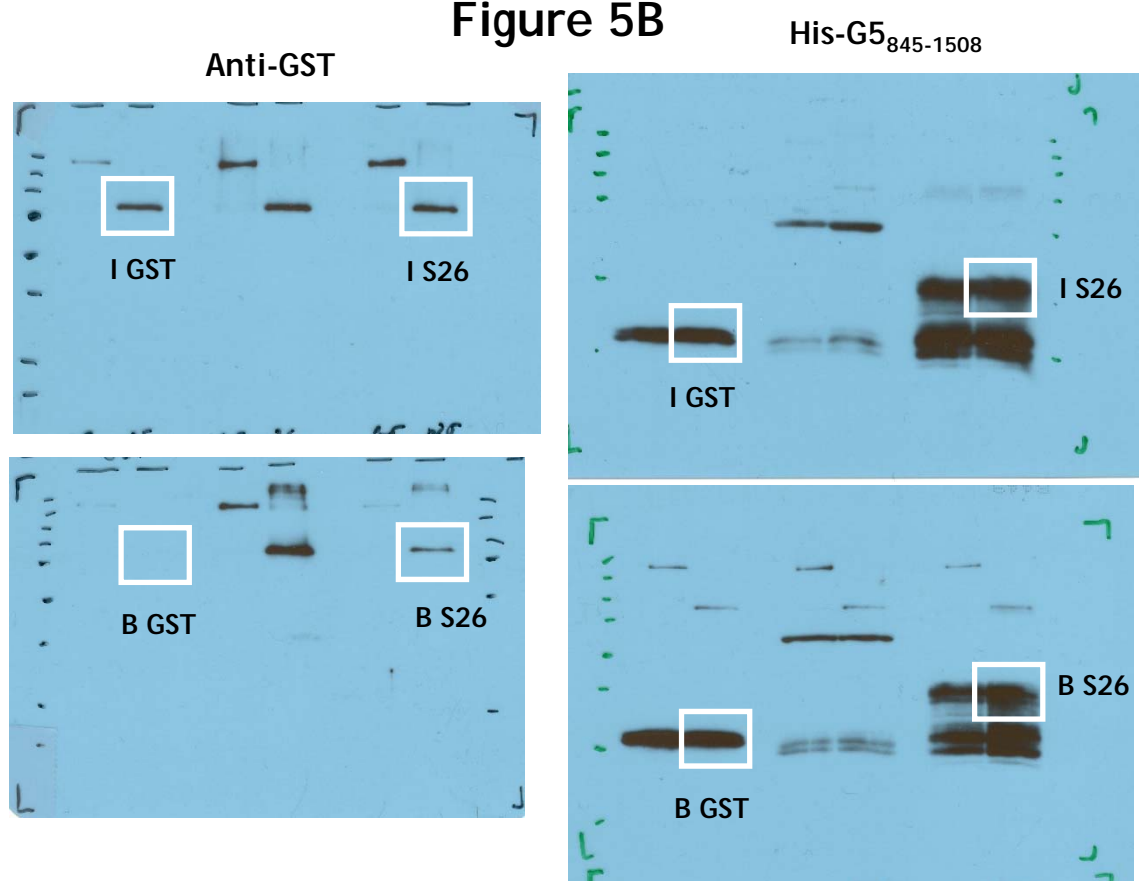

Figure 5B

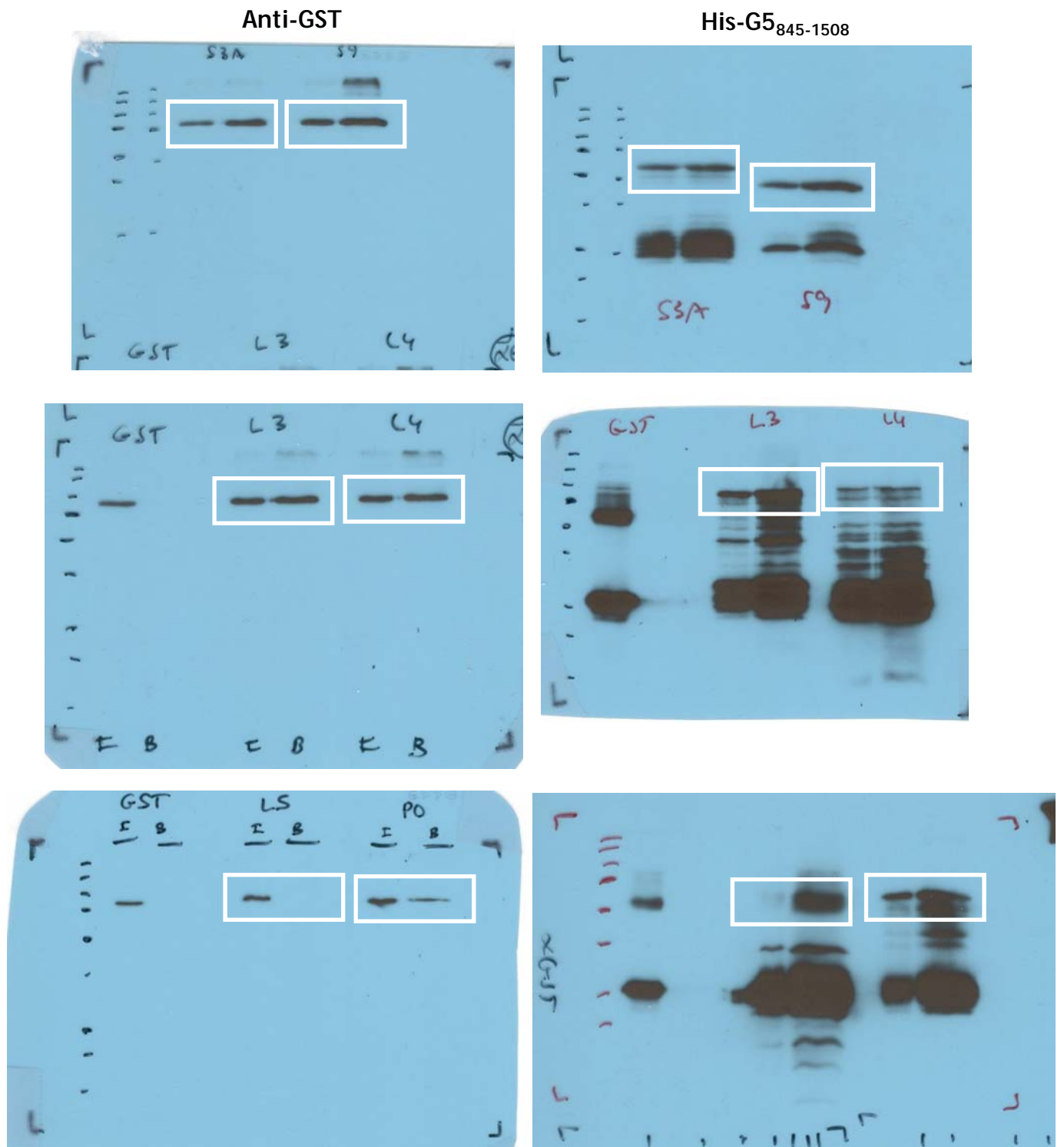

Figure 5D

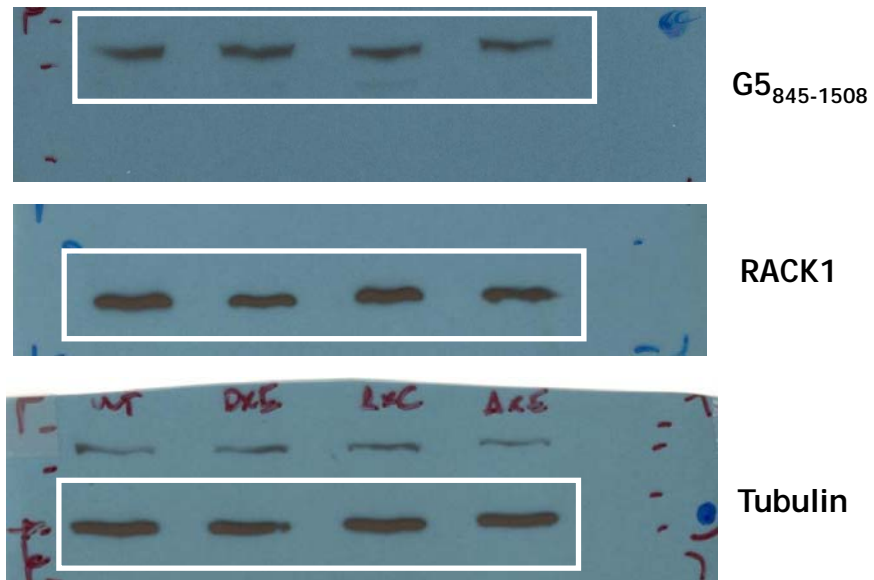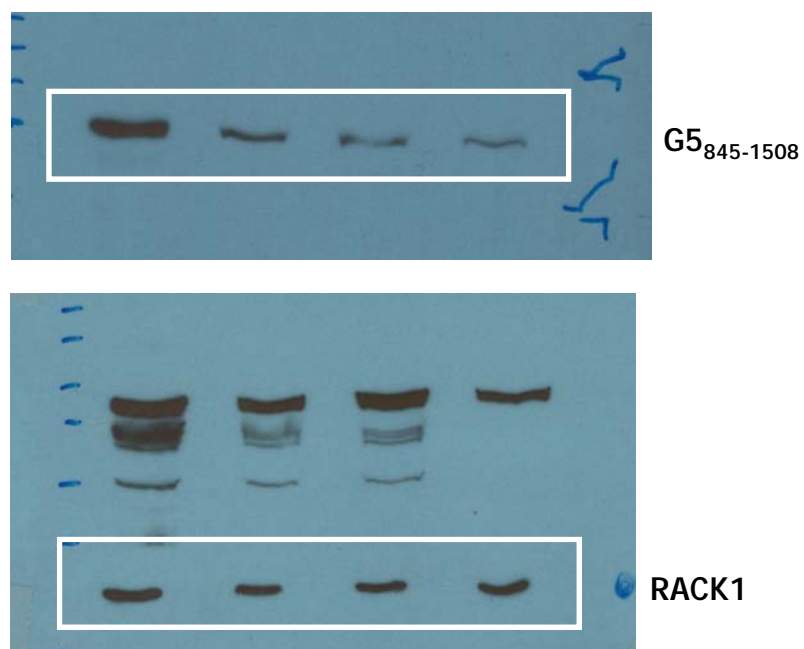

Supplement: Supplementary file 6 [file LSA-2022-01403_SdataF5.pdf]

Figure 6A

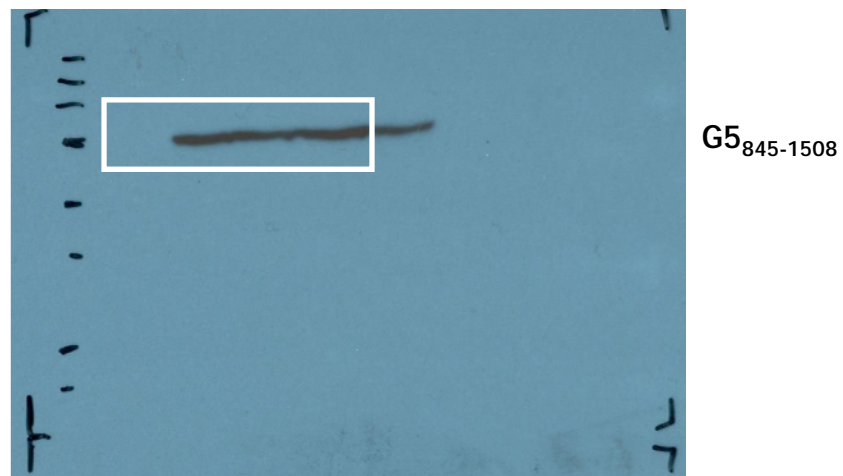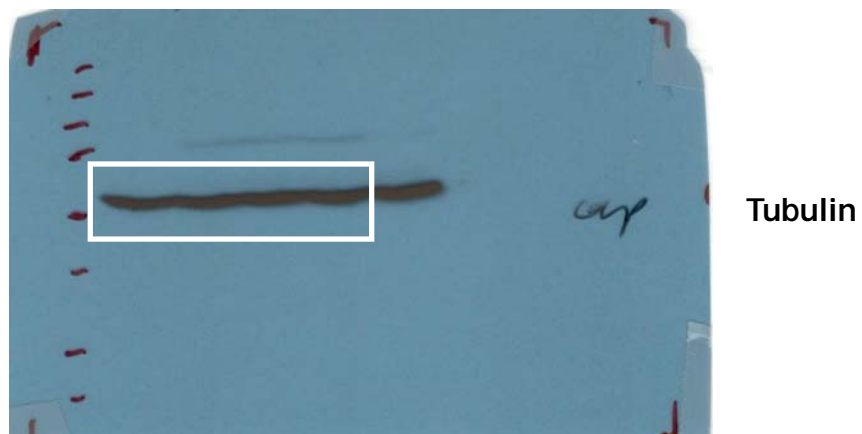

Figure 6C

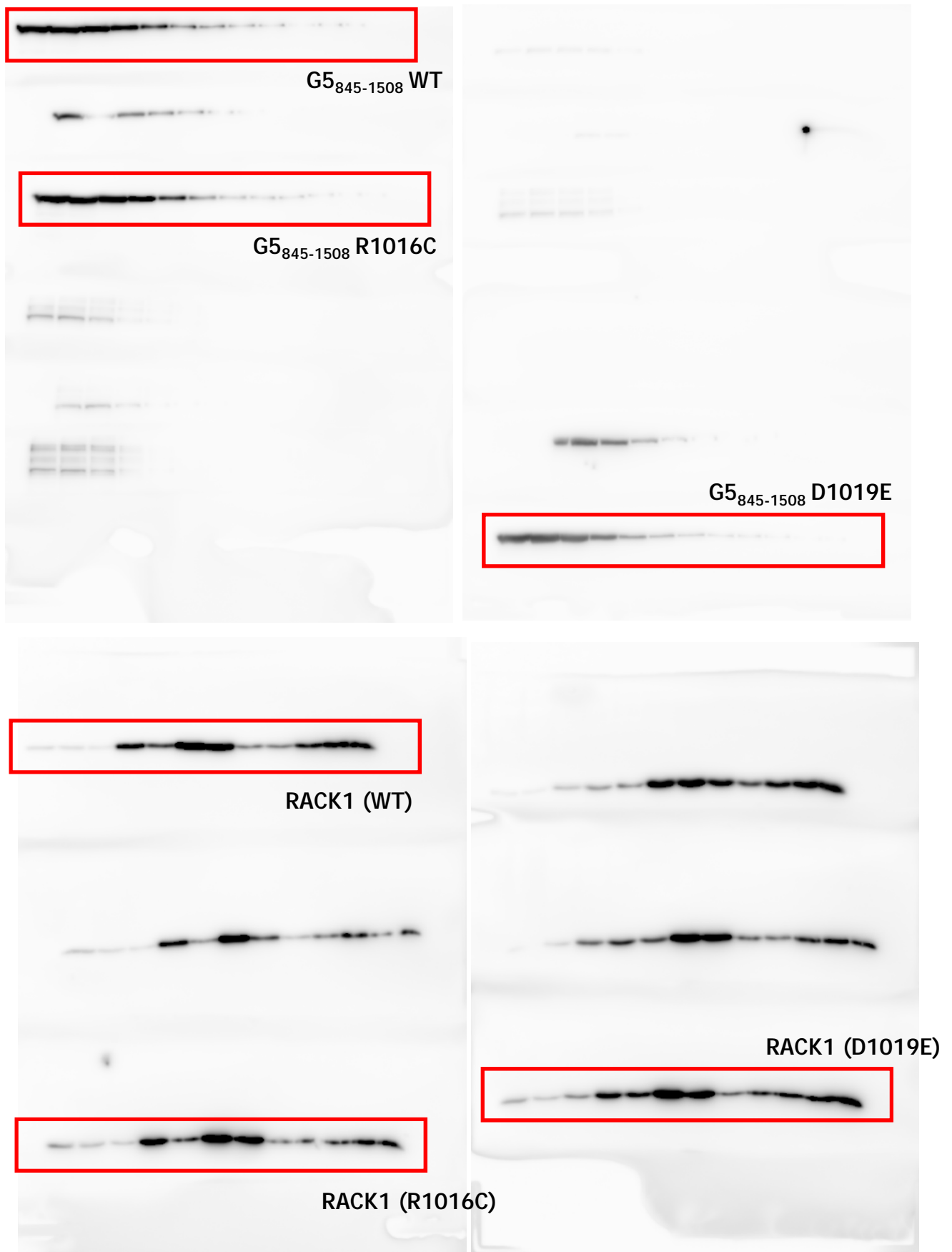

Supplement: Supplementary file 7 [file LSA-2022-01403_SdataF6.pdf]
