# Supplementary material for: Functional and structural deficiencies of Gemin5 variants associated with neurological disorders
Source: Life Sci Alliance. 2022 Apr 7;5(7):e202201403. doi: 10.26508/lsa.202201403 (PMC8989681; doi:10.26508/lsa.202201403)
Supplement: Supplementary file 8 [file LSA-2022-01403_TableS1.docx]

Table S1. Primers used in constructs and RTqPCR analysis.

| Oligo | Sequence (5´- 3´) |
| --- | --- |
| G5-D1019E s | tcaggactggctcctccgggcgc |
| G5-D1019E as | gcgcccggaggagccagtcctga |
| G5-R1016C s | gtcctccgggcacagccgggcct |
| G5-R1016C as | aggcccggctgtgcccggaggac |
| G5-L1367P s | gttctctgtgagttttggggactggcatgcttttctg |
| G5-L1367P as | cagaaaagcatgccagtccccaaaactcacagagaac |
| 5'EcoRI-GST-RPS9 | aagaattcacatgccagtgg |
| 3'SalI-GST-RPS9 | atgtcgacttaatcctcctcctcgt |
| 5'BamHI-RPS26 | taggatccatgacaaagaaaagaagg |
| 3'SalI-GST-RPS26 | atgtcgacttacatgggctttgg |
| His-Xpress s | ggggttctcatcatcatcatc |
| His-Xpress as | gatccttatcgtcatcgtcgt |
| AGO2-s | ataaagctattgcgacccctg |
| AGO2-as | tcagatggacttccgtgc |
| PCBP1-s | attcgccggaattgactcca |
| PCBP1-as | atggtgagttcatgggtggt |
| SF3B3-s | agaaatgttccagcccaccc |
| SF3B3-as | acttctccaggttcaaggcg |
| DHX15-s | ggtgtgtggagtacatgcga |
| DHX15-as | cagcaactctctgagccaca |
